# Novel Plasma Proteins Associated With Alzheimer’s Disease Risk in APOE ε4 Carriers and Non-carriers

**DOI:** 10.21203/rs.3.rs-10359931/v1

**Published:** 2026-08-03

**Authors:** Yuanming Leng, Huitong Ding, Jian Yang, Xue Liu, Alec Lu, Qiushan Tao, Jiantao Ma, Katherine A. Gifford, Ting Fang Alvin Ang, Wei Qiao Qiu, Rhoda Au, P. Murali Doraiswamy, Chunyu Liu

**Affiliations:** Boston University School of Public Health; Boston University Chobanian & Avedisian School of Medicine; Boston University School of Public Health; Boston University School of Public Health; College of Health & Rehabilitation Sciences: Sargent College, Boston University; Physiology & Biophysics, Boston University Chobanian & Avedisian School of Medicine; Friedman School of Nutrition Science and Policy, Tufts University; Boston University Chobanian & Avedisian School of Medicine; Boston University Chobanian & Avedisian School of Medicine; Physiology & Biophysics, Boston University Chobanian & Avedisian School of Medicine; Boston University Chobanian & Avedisian School of Medicine; Duke University School of Medicine; Boston University School of Public Health

**Keywords:** Alzheimer’s disease, proteomics, APOE, polygenic risk score, interaction, mediation

## Abstract

**Background:**

Whether plasma protein associations with Alzheimer’s disease (AD) differ by genetic risk remains unclear.

**Methods:**

We studied 18,212 UK Biobank participants aged ≥ 60 years with Olink Explore 3072 proteomic data, APOE genotypes, and AD polygenic risk scores. Protein-by-genetic risk interactions were screened, followed by genetic risk-stratified association and mediation analyses in Cox models.

**Results:**

During 12.8 years of follow-up, 374 participants developed AD. APOE ε4 carriers had higher AD risk than non-carriers (HR = 7.79 [6.18–9.81]), with the greatest risk among ε4 homozygotes (HR = 17.73 [13.36–23.51]). Stratified analyses identified AD-associated proteins among APOE ε4 non-carriers (n = 85), ε4 carriers (n = 10), and low-, intermediate-, and high-AD-PRS groups (n = 10, 1, and 2, respectively), including 94 newly identified proteins. APOE protein, MENT, and GFAP mediated 34.49%, 25.01%, and 11.47% of the APOE ε4-AD association.

**Conclusions:**

Genetic risk stratification revealed heterogeneous protein-AD associations and genotype-dependent proteomic pathways.

## Introduction

Alzheimer’s disease (AD) is a progressive neurodegenerative disorder[1]. Effective and affordable treatments remain limited[2], largely because AD is highly heterogeneous, with substantial variation in disease onset, progression, and treatment response[3]. This heterogeneity complicates efforts to define the molecular mechanisms underlying disease susceptibility. Genetic susceptibility is a major contributor to AD heterogeneity[4, 5]. The APOE ε4 allele is the strongest genetic risk factor for late-onset AD and has been linked to amyloid and tau pathology, neuroinflammation, and synaptic dysfunction[6]. Beyond APOE, AD polygenic risk scores (AD-PRS) derived from genome-wide association studies (GWAS) summarize the cumulative effects of multiple common genetic variants across biological pathways [7–11]. These genetic factors may influence the overall risk of developing AD, and also shape the molecular pathways through which AD risk emerges. Understanding whether circulating molecular markers differ across genetic risk strata may therefore provide important insight into genotype-dependent mechanisms of AD susceptibility.

High-throughput plasma proteomics platforms, such as Olink, offer a minimally invasive method to characterize thousands of proteins involved in biological processes relevant to AD. Several UK Biobank (UKB) studies using Olink 1536 and Olink 3072 platforms have identified plasma proteins associated with incident AD or dementia[12–20]. However, most prior studies have evaluated associations in the full sample. Although pooled analyses can identify proteins associated with AD across the overall population, they may obscure subgroup-specific associations that vary by genetic risk. A few studies have examined APOE-related heterogeneity, but evidence remains limited. For example, Zhang et al. used the Olink 3072 platform to evaluate protein associations with AD and protein-APOE ε4 interactions in the full sample, but no associations remained significant after multiple-testing correction[17]. Guo et al. performed APOE ε4-stratified analyses using the Olink 1536 platform and found limited evidence that APOE ε4 modify dementia-protein associations[15]. These studies provide important initial evidence but may have been underpowered to detect genetically modified protein associations. Prior work also has not comprehensively evaluated whether broader genetic risk, including both APOE ε4 carrier status, APOE ε4 homozygosity as a distinct high-risk subgroup[21], and AD-PRS, modifies plasma protein associations with incident AD.

To address this gap, we applied a two-stage analytical framework to evaluate whether plasma protein associations with incident AD differ across genetic risk strata defined by APOE ε4 carrier status, APOE ε4 homozygosity, and AD-PRS groups. Using Olink Explore 3072 proteomic data in UKB, in stage 1, we screened protein-genetic risk interactions. Proteins showing evidence of interaction were subsequently evaluated in stage 2 using stratified analyses within genetic risk strata. We further compared our findings with prior UKB plasma proteomic studies to assess whether genetic stratification identifies additional proteins that may be overlooked in pooled analyses. Finally, we evaluated whether selected AD-associated proteins mediated the association between APOE ε4 carrier status and incident AD.

## Methods

### Study Population

UKB is a large prospective cohort study that enrolled over 500,000 participants aged 39–70 years across the United Kingdom between 2006 and 2010[22, 23]. The cohort includes extensive baseline and longitudinal phenotypic, genetic, biomarker, and health outcome data. The present study focused on a subset of 53,014 participants with baseline plasma proteomic profiling measured using the Olink Explore 3072 platform [24]. We first excluded participants with self-reported dementia (n = 32), followed by those with prevalent dementia at baseline (n = 44), incident non-AD dementia during follow-up (n = 934), and those with less than one year of follow-up (n = 131). Participants with missing APOE genotype information (n = 442), those carrying the ε2/ε4 genotype (n = 1,350), or those with missing information on education (n = 555), were also excluded. To reduce the impact of missingness on proteomic analysis, we excluded participants with more than 20% missing values across the protein panel (n = 8,240). Lastly, individuals younger than 60 years at baseline (n = 23,074) were excluded. After applying all exclusion criteria, the final UKB sample consisted of 18,212 participants for subsequent analyses. Other UKB studies were presented in **Supplemental Tables 1 and 2.**

### Ethical Considerations

The UKB study was conducted in accordance with the Declaration of Helsinki and received ethical approval from the NHS National Research Ethics Service as part of the UKB’s general approval (June 17, 2011; Ref 11/NW/0382). All participants provided written informed consent. All data analyzed in this study were de-identified.

### Blood Proteomics

Baseline plasma proteomic profiling was performed using the Olink Explore 3072 platform, an antibody-based proximity extension assay (PEA)[25]. For each participant, a total of 2,923 plasma proteins were quantified and reported as normalized protein expression (NPX) values on a log2 scale[25, 26]. Proteins with more than 20% missing NPX values across participants were excluded, resulting in 2,911 proteins retained for analysis. The remaining missing NPX values were imputed using the protein-specific mean NPX value calculated across available samples.

### APOE ε4 Status

APOE genotype was derived from UKB whole-genome sequencing data. Single-nucleotide polymorphisms (SNPs) were extracted using PLINK v1.9[27]. APOE allelic status was determined based on two SNPs, rs429358 and rs7412, which jointly define the three common APOE alleles (ε2, ε3, and ε4)[28]. Because the ε2/ε4 genotype contains both the protective ε2 allele and the risk-associated ε4 allele, participants with this genotype were excluded from the primary analyses to avoid potential opposing allele effects[29]. In this study, APOE ε4 status was defined in two ways. First, participants were classified into a binary ε4 carrier variable: ε4 non-carriers included individuals with ε2/ε2, ε2/ε3, or ε3/ε3 genotypes, whereas carriers included individuals with ε3/ε4 or ε4/ε4 genotypes. Second, three APOE ε4 groups was modeled using a three-level categorical variable: non-carriers included ε2/ε2, ε2/ε3, and ε3/ε3; ε4 heterozygotes included ε3/ε4; and ε4 homozygotes included ε4/ε4.

### AD-PRS Derivation

This study included 25 non-APOE common SNPs (minor allele frequency [MAF] ≥ 0.05) previously reported in the GWAS literature to be associated with AD[30, 31] (**Supplemental Table 3**). The AD-PRS was calculated under an additive genetic model as the weighted sum of AD risk-increasing allele dosages across the selected SNPs. Risk alleles were aligned to the AD-increasing allele reported in prior GWAS, and SNP weights were defined using published GWAS effect-size estimates, corresponding to log-transformed hazard ratios (HRs) [30, 31]. Participants were then categorized into three AD-PRS groups: high (top quintile), intermediate (2nd −4th quintiles), or low (bottom quintile).

### Diagnosis of AD

AD status was ascertained using data from hospital admissions, primary care, and death registry records. The primary outcome was incident AD, defined using the UKB algorithmically derived AD event fields (fields 42018-25). Follow-up was calculated from the baseline assessment-center visit date (field 53) to the earliest of the following: the recorded date of diagnosis (field 42020), the date of death (field 40000), the date lost to follow-up (field 191), or the censor date, whichever occurred first. The censor date was adjusted based on the participant’s country (field 1647) and the source of the reported AD (field 42021), in line with the guidelines outlined in [32]. For countries not listed in the guideline, the default censoring date was set to November 30, 2022. A summary of the field ID for the key variables is provided in **Supplemental Table 4**.

### Statistical Analysis

#### Descriptive analysis of baseline characteristics

Baseline characteristics were summarized overall and by genetic subgroup. Continuous variables were described using means and standard deviations (SD). Categorical variables were summarized as counts and percentages. Baseline differences across genetic subgroups were evaluated using t-tests for continuous variables and Chi-square tests for categorical variables. Time-to-event patterns were examined using Kaplan-Meier curves to compare cumulative AD-free survival across genetic subgroups. Differences between survival curves were assessed using log-rank tests.

##### Associations of APOE ε4 carrier status and AD-PRS with incident AD:

**Figure 1:**
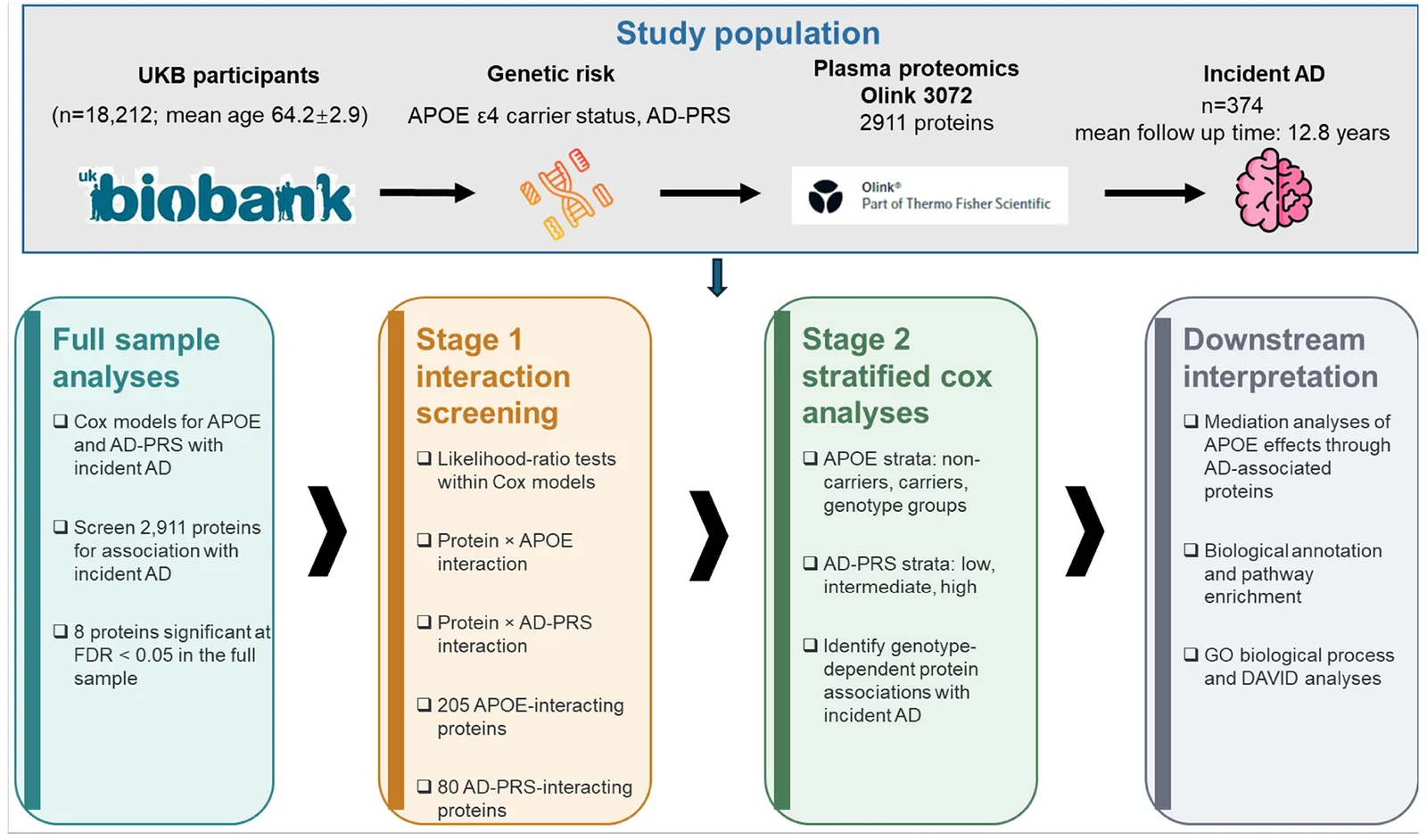
Study design and analytical workflow for genetic risk-stratified plasma proteomics of incident AD

We examined the associations of APOE ε4 carrier status and AD-PRS groups with incident AD (Fig. 1) using Cox proportional hazards models. These analyses were designed to estimate the total associations between these genetic risk factors and AD risk. Models were adjusted for baseline age, sex, education, and the first 20 genetic principal components (PCs). In models evaluating AD-PRS group as the primary predictor, APOE ε4 carrier status was additionally included as a covariate. Effect estimates were reported as HRs [95% confidence intervals (CIs)]. The model was specified as: **Model 1**: IncidentAD∼APOEε4carrierstatus/AD-PRSgroup+covariates.

#### Associations of plasma proteins with APOE ε4 carrier status and AD-PRS

We examined whether genetic risk factors were associated with circulating protein levels using linear regression models. In these models, each plasma protein was analyzed separately as the outcome, and APOE ε4 carrier status or AD-PRS level was modeled as the primary independent variable. The same covariate adjustment was used as described above. The model was specified as

Model 2 Proteinlevel∼APOEε4carrierstatus/AD-PRSlevel+covariates

Proteins with an FDR < 0.05 after correction for all proteins were regarded as statistically significant.

##### Associations between plasma proteins and incident AD in the full sample:

Each plasma protein was examined separately as the primary exposure using Cox proportional hazards models. Incident AD was modeled as the time-to-event outcome. **Model 3**: lncidentAD∼proteinlevel+APOEε4carrierstatus+covariates We further fitted a sensitivity model, additionally adjusted for AD-PRS group. Proteins associated with incident AD at false discovery rate (FDR) < 0.05 after correction for all proteins were considered statistically significant (Fig. 1).

#### Interaction and stratified analyses by genetic risk

We evaluated whether associations between plasma proteins and incident AD differed by genetic risk. Genetic risk was defined using binary APOE ε4 carrier status, three APOE ε4 groups, and the AD-PRS group. We first tested interactions in the full sample. For each protein, we fitted a Cox proportional hazards model that included the protein, genetic risk group, and their interaction term with covariate adjustment as described above.

Model 4 incidentAD∼proteinlevel+geneticriskgroup+proteinlevel×geneticriskgroup+covariates

Likelihood ratio tests (LRTs) were used to compare models with and without the interaction term. Proteins with LRT *P* < 0.05 were identified as candidates for stratified analyses. These analyses were intended to identify proteins whose associations with incident AD may vary across genetic risk strata. For candidate proteins, we then estimated protein-AD associations within strata defined by: binary APOE ε4 carrier status (non-carriers and carriers), three APOE ε4 groups (non-carriers, heterozygotes, and homozygotes), and AD-PRS group (low, intermediate, and high risk). Within each stratum, we fitted Cox proportional hazards models:

Model 5 incidentAD∼proteinlevel+covariates

Statistical significance was defined as FDR < 0.05, with correction based on the number of candidate proteins identified from the interaction screening step (Model 4).

#### Mediation analyses of plasma proteins linking APOE ε4 carrier status to incident AD

We conducted mediation analyses to assess whether specific plasma proteins may mediate the association between APOE ε4 carrier status and incident AD. Candidate mediators were selected from proteins that were significantly associated with incident AD in the full sample in Model 3 at FDR < 0.05 (Fig. 1). For each candidate protein, mediation estimates were derived using a mediator model for protein level as a function of APOE ε4 carrier status and covariates, and an outcome model for incident AD as a function of APOE ε4 carrier status, protein level, and covariates. We estimated the natural direct effect (NDE), the natural indirect effect (NIE), and the proportion mediated by integrating results from Model 2 and Model 3. Effects were expressed on the hazard-ratio scale. Statistical uncertainty was assessed using 1,000 bootstrap resamples to derive 95% CIs and P values.

##### Cumulative AD risk curves:

To illustrate the absolute cumulative incidence of AD over time across AD genetic risk subgroups and contrasting protein expression levels, the standardized predicted cumulative AD risk curves were derived using Cox proportional hazards models, stratified by APOE ε4 carrier status and adjusted for age, sex, education, and 20 genetic PCs. Protein levels were categorized as “low” and “high” using the interquartile range (IQR: Q3-Q1) method: low protein level was defined as Q1–1.5 * IQR, and high as Q3 + 1.5 * IQR, with Q1 and Q3 indicating the first and third quartiles. Calibration adjusted the baseline hazard to match the external population-level incidence rate for AD (11.98 per 10,000 person-years)[33]. This calibration aligned predicted risk with real-world disease frequency while maintaining the relative protein effects estimated by the Cox model.

##### Enrichment analysis:

To investigate the biological functions represented by AD-related proteins, we performed Gene Ontology (GO) enrichment and curated pathways using the over-representation analysis (ORA) approach[34]. Separate enrichment analyses were performed for proteins identified in the stratified analysis by APOE ε4 status and AD-PRS groups in UKB. Enrichment significance was determined based on two criteria: FDR *P* < 0.05 and enrichment score greater than 1. To reduce redundancy among overlapping GO terms, semantic similarity was calculated, and terms were grouped into related clusters. Analyses are conducted by the clusterProfiler R package[34, 35].

## Results

### Cohort characteristics

The study included 18,212 UKB participants aged 60 and older (mean age: 64.2 ± 2.9 years, 52.9% women) (Table 1), among whom 374 (2.1%) developed AD over a mean follow-up period of 12.8 years. APOE ε4 carriers (26.6% of the cohort) exhibited a higher incidence of AD than non-carriers (5.7% vs. 0.7%, *P* < 0.001), despite similar distributions of age, sex, and education (*P* > 0.05 for all). Within carriers, the APOE ε4 homozygous group (3.4% of the cohort) exhibited a higher incidence of AD than the APOE ε4 heterozygous group (23.2% of the cohort) (16.6% vs. 4.1%, *P* < 0.001). Similarly, AD incidence increased across AD-PRS groups: 1.5%, 2.0%, and 2.8% in the low, intermediate, and high groups, respectively (*P* < 0.001). Participants in AD-PRS groups differed modestly by sex (range: 51.7%-53.7% women, *P* = 0.03), while age and education remained balanced across these strata (Table 1).

### Associations of APOE ε4 carrier status and AD-PRS groups with AD

**Figure 2:**
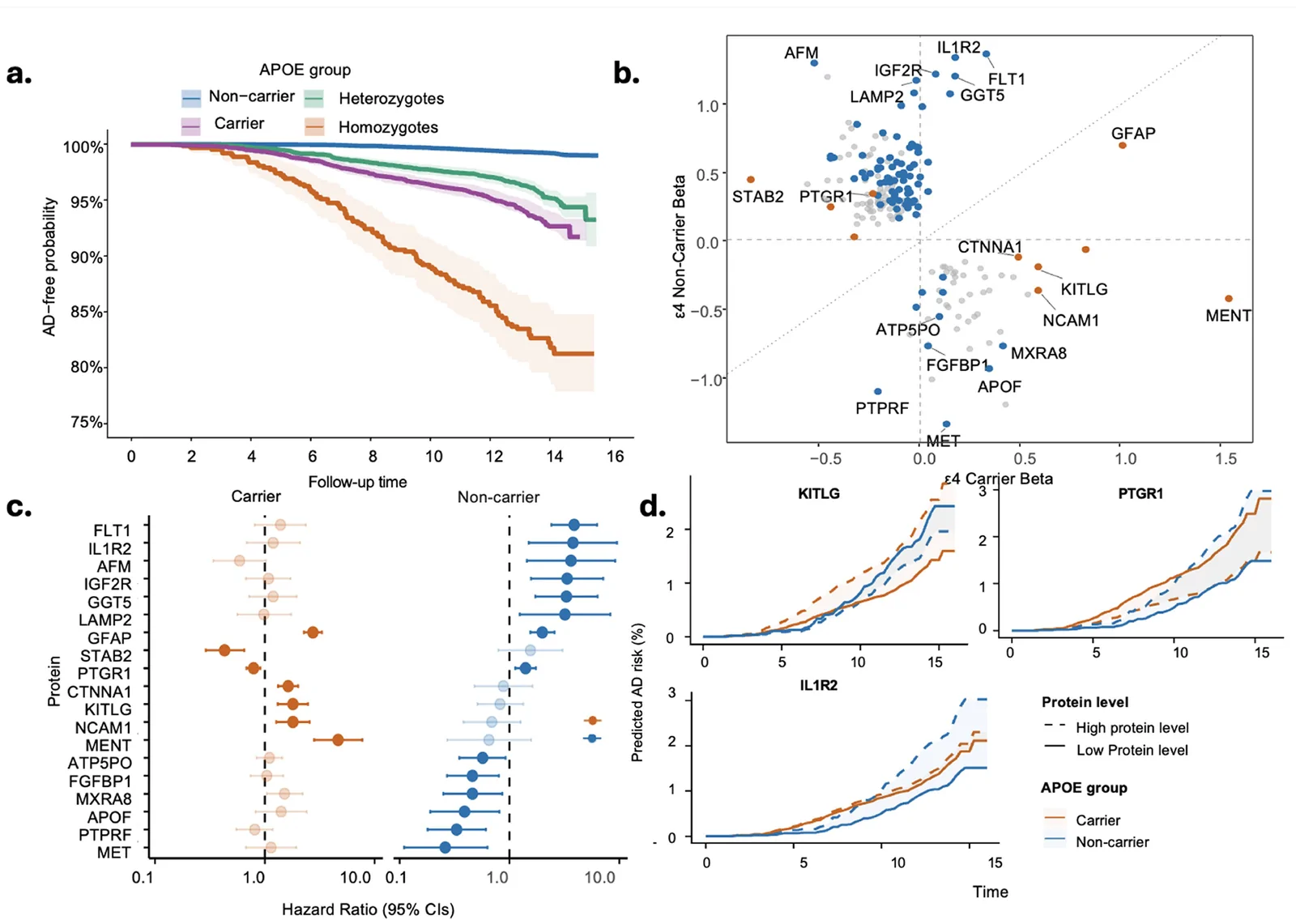
APOE ε4–specific plasma protein associations of Alzheimer’s disease risk. a. Kaplan-Meier curves of AD-free survival stratified by APOE ε4 genotype. b. Differential protein-AD associations by APOE ε4 carrier status. This scatter plot compares the estimated associations between 205 plasma proteins and incident AD stratified by APOE ε4 carrier status. The x-axis shows the cox model β coefficients among APOE ε4 non-carriers, and the y-axis shows the corresponding cox model β coefficients among APOE ε4 carriers. Each point represents one protein. Points highlighted in blue or red indicate proteins reaching adjusted significance in non-carriers or carriers, respectively. Gray points indicate proteins that did not reach adjusted significance in either group. Two proteins, GFAP and PTGR1, that are associated with incident AD in both groups were colored green. The top six newly identified proteins with the strongest positive and negative associations among APOE ε4 non-carriers, along with all five newly identified proteins among APOE ε4 carriers were labeled. Two proteins that were significantly associated with incident AD in both groups (GFAP and MENT) were also labeled. c. Forest plot of HRs and 95% CIs for 19 selected proteins including top six proteins with the strongest positive and negative associations among APOE ε4 non-carriers, all five newly identified proteins among APOE ε4 carriers, and two proteins (GFAP and MENT) that were associated with incident AD in both groups. d. Calibrated standardized predicted cumulative AD risk by protein level and APOE ε4 carrier status. KITLG, PTGR1, and IL1R2 were shown as example proteins. Standardized predicted cumulative AD risk curves were generated from Cox proportional hazards models stratified by APOE ε4 carrier status and adjusted for age, sex, education, and the first 20 genetic PCs. For each protein, predicted risks were estimated at prespecified low and high protein levels, defined as Q1 – 1.5 × IQR and Q3 + 1.5 × IQR, respectively. To estimate absolute cumulative risk, the model-based baseline hazard was calibrated to an external population-level AD incidence rate of 11.98 per 10,000 person-years at the calibration horizon, while preserving the relative effects estimated from the Cox model.

AD-free Kaplan-Meier curves showed clear separation by both APOE ε4 carrier status and AD-PRS groups (Fig. 2a and **Supplementary Fig. 1**). APOE ε4 carriers had lower AD-free survival than non-carriers (log-rank test *P* < 0.001). After adjustment for age, sex, and education, APOE ε4 carrier status remained strongly associated with incident AD (HR = 7.79 [6.18–9.81], **Supplemental Table 5: Model c**). When carriers were further stratified by ε4 allele dose, APOE ε4 homozygotes had the highest risk relative to non-carriers (HR = 17.73 [13.36–23.51]), followed by heterozygotes (HR = 5.87 [4.58–7.53], **Supplementary Table 5: Model d**). For PRS groups, participants in the highest PRS quintile had higher AD risk than those in the lowest quintile (HR = 1.83 [1.32–2.54]), whereas the intermediate quintiles (2nd-4th) did not differ significantly (HR = 1.27 [0.95–1.71]; **Supplementary Table 5: Model b**). When APOE ε4 status and AD-PRS were modeled jointly, the effect estimates were largely similar, suggesting largely independent contributions of APOE ε4 and AD-PRS to AD risk (**Supplemental Table 5: Model a**).

### Proteins associated with APOE ε4 status and AD-PRS groups

Among the 2,911 proteins analyzed, 343 proteins were significantly associated with APOE ε4 carrier status (FDR < 0.05; **Supplemental Table 6**) after adjusting for age, sex, education, and 20 genetic PCs. Using APOE ε4 non-carriers as the reference, 296 proteins showed lower plasma levels in ε4 carriers, with effect estimates ranging from − 0.64 to −0.01. For example, plasma APOE levels were, on average, 0.64 NPX units lower among ε4 carriers compared with non-carriers. In contrast, 47 proteins showed higher plasma levels among ε4 carriers, with effect estimates ranging from 0.01 to 0.33. Among these, SNAP25 showed the largest effect size, with plasma levels 0.33 NPX units higher in ε4 carriers than in non-carriers.

Many fewer proteins were associated with AD-PRS levels. After adjusting for age, sex, education, and 20 genetic PCs, 17 proteins were significantly associated with AD-PRS levels (FDR < 0.05; **Supplemental Table 7**). Nine proteins showed lower levels with higher AD-PRS levels, with effect estimates ranging from −0.04 to −0.21. PIRLB exhibited the largest decrease, with plasma levels 0.21 NPX units lower per unit increase in AD-PRS. In contrast, eight proteins showed higher plasma levels as AD-PRS increased, with effect estimates ranging from 0.04 to 0.13. CD2AP plasma levels increase by 0.13 NPX units, the largest increase, for each unit increase in AD-PRS.

### Proteins associated with incident AD in the full sample

Associations between 2,911 plasma proteins and incident AD were examined in the full sample after adjustment for age, sex, education, APOE ε4 carrier status, and 20 genetic PCs. Eight proteins were associated with incident AD at FDR < 0.05: GFAP, NEFL, SYT1, CEND1, TGFBR1, APOE, MENT, and BCAN (Table 2). All eight have been reported for their association with incident AD or dementia and showed concordant directions of association in our study and prior reports [13, 15–20]. For example, each NPX unit increase in its plasma APOE was associated with a 43% lower risk of incident AD (HR = 0.57 [0.44, 0.73]). After adjusting for AD-PRS level, the associations of all proteins with AD risk remain largely unchanged (**Supplemental Table 8**).

### Stratified protein associations with incident AD by APOE ε4 carrier status

We identified 205 proteins showing nominal evidence of interactions with APOE ^4 carrier status (LRT *P* < 0.05). These proteins were then evaluated for their associations with incident AD within APOE ε4 strata, first comparing ε4 non-carriers **with** ε4 carriers and then further stratifying carriers into heterozygous and homozygous carriers. Among APOE ε4 non-carriers, 85 of the 205 proteins were significantly associated with incident AD at FDR < 0.05 (Fig. 2b, **Supplementary Table 9**). Of these, six proteins have been reported previously in UKB full sample analyses: KRT19 [13], LGALS4 [13, 18], TNFRSF11A[13], COL6A3[13, 15], HGF[18], and GFAP[13, 15–17, 19, 20]. The remaining 79 proteins represented novel associations. Ten of them were inversely associated with AD risk, with HRs ranging from 0.26 to 0.76. Among these, MET showed the strongest inverse association (HR = 0.26 [0.11, 0.63]). The other 69 newly identified proteins were positively associated with AD risk, with HRs ranging from 1.17 to 3.88. FLT1 showed the largest effect estimate (HR = 3.88 [2.41, 6.27]) (Fig. 2c).

Among APOE ε4 carriers, 10 of 205 proteins were associated with incident AD at FDR < 0.05. (Fig. 2b, **Supplementary Table 10**). These included 5 proteins previously identified in non-stratified analyses, GFAP [13, 15–17, 19, 20], MENT[19, 20], DSG2[13, 15], LDLR[13, 15, 17, 19], and GPC5[13, 15], together with 5 newly identified proteins. Among these newly identified markers, higher levels of NCAM1, KITLG, and CTNNA1 were associated with increased AD risk (NCAM1: HR = 1.80 [1.27–2.56, KITLG: HR = 1.80 [1.31–2.47], CTNNA1: HR = 1.63 [1.32–2.01]), whereas PTGR1 and STAB2 were inversely associated with AD risk (PTGR1: HR = 0.79 [0.68–0.91]; STAB2: HR = 0.43 [0.29–0.65]) (Fig. 2c). Subsequent analyses were conducted among ε4 carriers to assess how ε4 allele numbers modify the association between 205 proteins and AD risk, identifying 5 proteins in heterozygous (ε3/ε4) individuals and 1 protein in homozygous (ε4/ε4) individuals. Among the five proteins identified in heterozygous individuals, CTNNA1 and LDLR showed effect sizes similar to those observed in all carriers. Two proteins were additionally identified in heterozygous individuals, with the RET protein decreasing the risk of AD incidence (HR = 0.56 [0.40–0.79]) and the CUZD1 protein increasing AD risk (HR = 1.66 [1.24–2.23]) (**Supplemental Table 11**).

Figure 2d shows representative APOE ε4-stratified predicted cumulative AD risk curves illustrating heterogeneity in protein-AD associations by genetic background (**Supplemental Fig. 2**). For KITLG, which was identified among APOE ε4 carriers, higher protein levels were associated with higher predicted cumulative AD risk in ε4 carriers, whereas the corresponding risk curves among non-carriers showed an opposite pattern. For PTGR1, stratified analyses suggested opposing directions of association by APOE ε4 carrier status, with higher levels associated with lower predicted AD risk in carriers but higher predicted risk in non-carriers. This opposing pattern may explain why PTGR1 was not detected in the full sample analysis. For IL1R2, higher protein levels were associated with greater predicted cumulative AD risk primarily among APOE ε4 non-carriers, with minimal separation by protein level among carriers.

### Proteins associated with incident AD by AD-PRS groups

For interaction with AD-PRS, 80 showed evidence of effect modification (LRT P < 0.05) across PRS strata (**Supplemental Fig. 3**). In the low AD-PRS group, 10 proteins were significantly associated with incident AD at FDR < 0.05 (**Supplemental Table 12**). Of these, two proteins (CALB1 and CHI3L1) have been previously reported to be associated with AD risk, whereas the remaining eight were novel findings. Among 8 novel proteins, two proteins were inversely associated with AD risk: C1QL2 (HR = 0.34 [0.17, 0.67]) and TEF (HR = 0.35 [0.17, 0.74]). The remaining six proteins were positively associated with AD risk, with HRs ranging from 1.37 to 6.11. Among the significant proteins, C1S showed the largest effect size (HR = 6.11 [1.80, 20.71]). In the intermediate AD-PRS group, CALB1 was the only one significantly associated with AD risk (HR = 1.67 [1.27, 2.19]), consistent with previously reported findings (**Supplemental Table 13**). In the high AD-PRS group, two newly identified proteins were associated with increased AD risk: CBX2 (HR = 1.61 [1.21, 2.15]) and PRR5 (HR = 1.72 [1.28, 2.30]) (**Supplemental Fig. 4, Supplemental Table 14**).

### Protein mediators of APOE on incident AD

**Figure 3:**
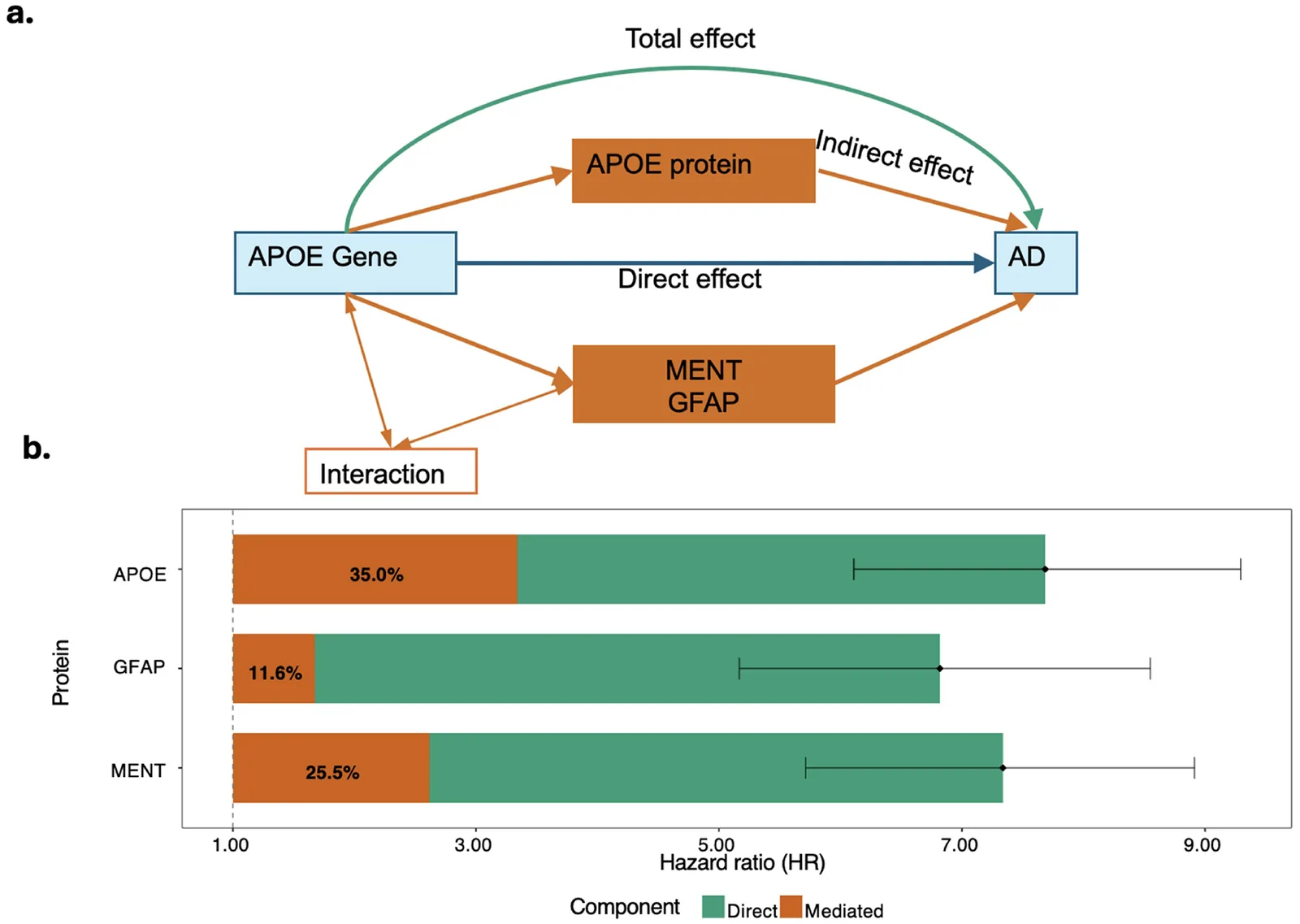
Mediation of the association between APOE ε4 carrier status and incident AD by selected proteins. Three proteins, including APOE, GFAP, and MENT, with proportional mediation estimates greater than 10% were selected. a. Directed acyclic graph illustrating the conceptual mediation framework, in which APOE ε4 carrier status influences incident AD risk through both direct and indirect pathways mediated by circulating proteins. b. Decomposition of the total effect of APOE ε4 carrier status on incident AD into direct and protein-mediated components. Labels indicate the estimated proportion mediated for each protein. Error bars represent 95% CI for HR of total effect.

Mediation analysis was performed for the 8 proteins associated with AD in full samples (FDR < 0.05) (**Supplementary Table 15**). The total effect of APOE ε4 on AD was HR = 7.79 [6.18–9.81]. Decomposition of the total effect of APOE ε4 carrier status on incident AD into direct and protein-mediated components, where each protein was evaluated as a single mediator, 6 of 8 showed a significant natural indirect effect (NIE) after FDR correction (**Supplementary Table 16**). Three proteins mediated over 10% of the total APOE ε4 effect on AD risk: APOE protein 35.02% [13.26–52.66%], MENT 25.53% [17.96–32.51%], and GFAP 11.58% [8.76–15.08%] (Fig. 3).

### Biological annotation of proteins associated with AD in stratified analyses

**Figure 4:**
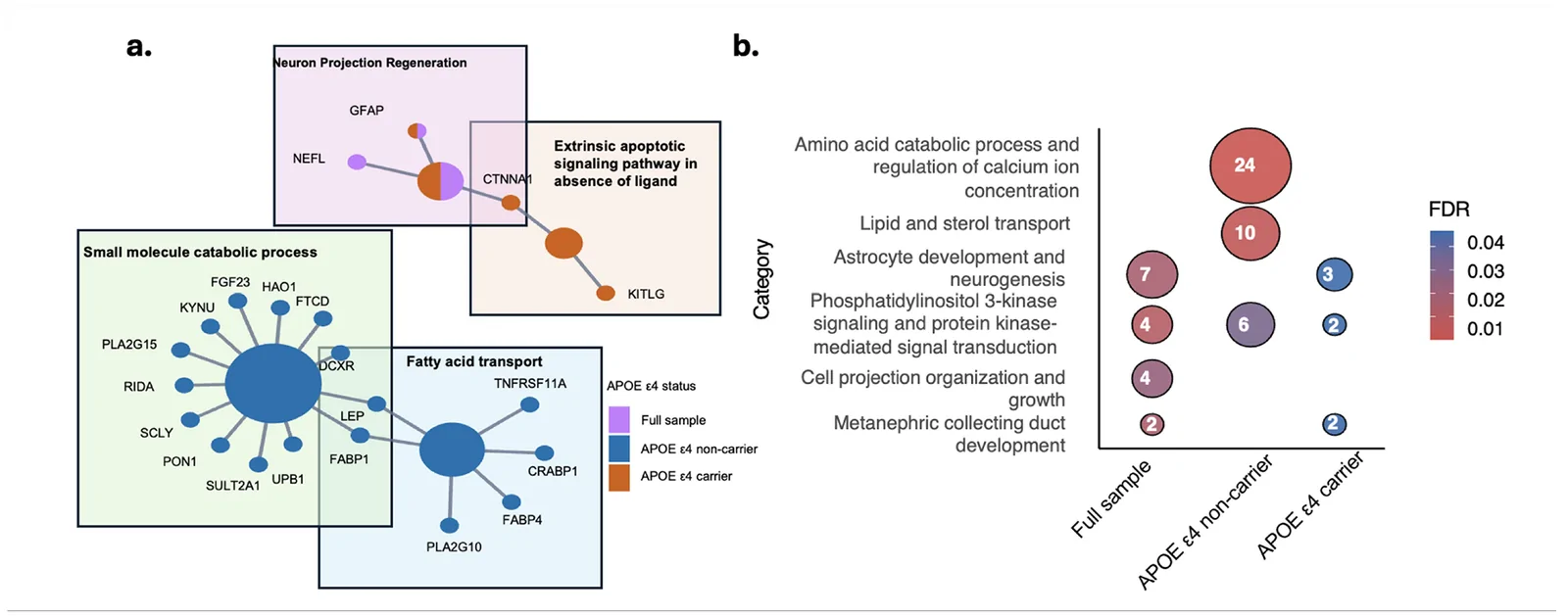
Enrichment analysis of AD-associated proteins by APOE ε4 status. a. Pathway enrichment across the full sample and APOE ε4-stratified subgroups. This bubble plot summarizes enriched biological pathways identified in the full sample, APOE ε4 non-carriers, and APOE ε4 carriers, after grouping similar enriched terms into GO-style functional categories. Bubble size represents the number of enriched proteins within each functional category and subgroup, and bubble color represents the FDR-adjusted *P* value. b. Newly identified network linking proteins to enriched GO processes. Node size reflects the number of mapped proteins contributing to each process, and colors indicate the stratum in which the signal was prioritized (full sample, ε4 non-carrier, or ε4 carrier)

Stratification by APOE ε4 status revealed distinct enrichment patterns among AD-associated proteins (FDR < 0.05). Among APOE ε4 non-carriers, associated proteins were enriched for small molecule catabolic process (GO: 0044282), cellular response to extracellular stimulus (GO: 0031668), and fatty acid transport (GO: 0015908). Newly identified proteins in this stratum showed similar enrichment for small molecule catabolic process and fatty acid transport. In contrast, among APOE ε4 carriers, associated proteins were enriched for neuron projection regeneration (GO: 0031102), ovarian follicle development (GO: 0001541), and the extrinsic apoptotic signaling pathway in the absence of ligand (GO: 0097192). (Fig. 4.b). The newly identified proteins in APOE ε4 carriers, CTNNA1 and KITLG, were specifically enriched for the extrinsic apoptotic signaling pathway in the absence of ligand (Fig. 4.a, **Supplemental Table 16**).

Stratification by AD-PRS also revealed distinct enrichment patterns among AD-associated proteins. In the low AD-PRS group, the newly identified proteins (CGA and GAS2) contributed to the enrichment of the rhythmic process pathway (GO:0048511) and ovarian follicle development (GO:0001541). In the high AD-PRS group, the newly identified protein CBX2 and PRR5 contributed to enrichment of the development of primary sexual characteristics (GO:0045137), positive regulation of cell growth (GO:0030307), and TORC2 signaling (GO:0038203) (**Supplemental Fig. 5 and Supplemental Table 16**).

## Discussion

In this large population-based cohort study, we systematically evaluated whether associations between plasma proteins and incident AD differed across genetic risk defined by APOE ε4 carrier status, APOE ε4 allele dose, and AD-PRS. Using a two-stage analytic framework, we identified heterogeneous plasma proteomic associations with incident AD. We identified 106 proteins associated with AD in genetic risk-stratified analyses that were not detected in the full sample analysis, and 94 of these are not reported in the UKB cohort. These findings support the importance of accounting for genetic background when evaluating circulating protein markers and biological pathways related to AD risk.

A major implication of this study is that APOE ε4 status may modify both the magnitude and direction of protein-AD associations. APOE ε4 is a strong genetic risk factor for AD[36], but our findings suggest that its relevance extends beyond risk prediction alone. The biological context of APOE ε4 carrier status appears to be reflected in differences in the circulating proteins associated with AD risk. Some proteins showed associations primarily among APOE ε4 non-carriers, whereas others were more evident among carriers. In certain cases, protein associations appeared to move in opposite directions across APOE ε4 strata. This provides a potential explanation for why these proteins were not identified in pooled analyses. This observation supports the broader concept that AD may arise through partially distinct molecular pathways depending on genetic background[37].

The results also highlight the value of moving beyond full sample proteomic association analyses. Prior plasma proteomic studies in AD have largely focused on identifying proteins associated with disease risk in the overall population[15, 17]. Such analyses are useful for detecting robust protein markers across populations, such as GFAP, NEFL, APOE, and other proteins repeatedly identified for AD or dementia[15, 38]. However, full-sample analyses may miss associations that are specific to genetic-risk subgroups. Our two-stage framework helped identify protein-AD associations within specific genetic subgroups that may be masked in full sample analyses. This approach may help partially explain inconsistencies across prior studies, including differences in the direction or magnitude of reported protein associations. As an example, protein PTGR1 is not associated with AD in the overall sample, but it shows a negative association with AD among APOE ε4 carriers and a positive association with AD in non-carriers (**Supplemental Table 11**). In contrast, other proteins appeared more subgroup-specific, suggesting that different biological pathways may be more relevant among APOE ε4 carriers versus non-carriers. These findings support the need to interpret plasma protein biomarkers in relation to the genetic background. Further stratification of the APOE ε4 carrier group, only GFAP remained significant among APOE ε4 homozygotes, providing limited evidence of homozygote-specific associations. GFAP may reflect both shared and genetically modified AD biology, as its association with AD was observed across genetic strata but appeared stronger among individuals with greater APOE ε4 burden. This pattern is consistent with the possibility that astrocytic activation or neuroinflammatory processes are broadly relevant to AD but may be amplified in the context of APOE ε4-related susceptibility[39, 40].

AD-PRS stratification provided additional evidence that genetic background beyond APOE may contribute to proteomic heterogeneity in AD. The number of significant proteins identified across AD-PRS strata was smaller than in APOE-stratified analyses, likely reflecting the stronger effect of APOE ε4. However, the presence of PRS-stratum-specific protein associations suggests that broader genetic susceptibility may influence circulating protein profiles related to AD. Future studies using larger samples may better characterize how polygenic architecture modifies proteomic pathways involved in AD.

The mediation findings further suggest that selected plasma proteins may partially capture biological processes linking APOE ε4 to AD risk. APOE protein, MENT, and GFAP each mediated more than 10% of the association between APOE ε4 carrier status and incident AD. However, these findings should be interpreted cautiously. For APOE protein, the mediation signal may largely reflect between-genotype differences in circulating APOE levels rather than a within-stratum protein effect. By contrast, GFAP showed evidence of both mediation and APOE-dependent differences in association magnitude. This supports its potential role as a marker of genotype-related neuroinflammatory or astrocytic pathways.

The pathway enrichment analyses also support the presence of genetically distinct proteomic patterns. Proteins associated with AD among APOE ε4 non-carriers were enriched for processes related to metabolism, extracellular response, and fatty acid transport, whereas proteins associated with AD among APOE ε4 carriers were enriched for processes related to neuronal regeneration and apoptotic signaling. Although these findings are exploratory, they suggest that different biological processes may underline AD risk across genetic strata. This may be particularly important for biomarker development, because proteins that perform well as risk markers in one genetic subgroup may not have the same relevance in another.

This study has several strengths. It used a large prospective cohort with long follow-up, high-throughput plasma proteomic profiling, APOE genotype, AD-PRS, and clinical AD diagnosis. The two-stage interaction and stratified analysis framework allowed us to move beyond average protein effects and identify potential genotype-dependent associations. In addition, the integration of Cox models, cumulative risk curves, mediation analyses, and pathway annotation provided complementary evidence supporting genetic heterogeneity in protein-AD associations. Several limitations should be noted. First, although the overall sample size was large, stratified analyses reduced the number of participants and AD events within subgroups, particularly among APOE ε4 homozygotes and high AD-PRS participants. Therefore, some subgroup-specific estimates may require replication. Second, AD outcomes were based on clinical diagnoses rather than AT(N) biomarker-defined disease, which is characterized by amyloid, tau, and neurodegeneration biomarkers. Third, circulating protein concentrations may be subject to confounding by factors such as body mass index, estimated glomerular filtration rate, and other metabolic or inflammatory conditions. Fourth, mediation analyses were observational and should not be interpreted as evidence of causal pathways. Fifth, replication of independent and more diverse cohorts is needed. Finally, residual confounding and measurement variability in proteomic data cannot be fully excluded.

In conclusion, our findings demonstrate that genetic stratification reveals substantial heterogeneity in plasma protein associations with incident AD. APOE ε4 carrier status, APOE ε4 allele dose, and AD-PRS may shape both the magnitude and direction of protein-AD associations. These results highlight the importance of accounting for genetic background in proteomic studies of AD and identify potential genotype-dependent biological pathways.

## Supplementary Material

This is a list of supplementary files associated with this preprint. Click to download.

- SuppFigure0710.docx
- SuppTableProteinGeneticUKB.xlsx

## Figures and Tables

**Table 1:** Baseline characteristics of 18,212 UK Biobank participants aged ≥ 60 years, by APOE ε4 carrier status and AD PRS group

Variable	Total (n = 18,212)	APOE ε4 carrier status			PRS			
		Non-carrier (n = 13,364)	Carrier (n = 4,848)	*P* value	Low (n = 3,643)	Intermediate (n = 10,926)	High (n = 3,643)	*P* value
Age	64.2 (2.9)	64.2 (2.9)	64.3 (3.0)	0.06	64.2 (2.9)	64.2 (2.9)	64.3 (2.9)	0.68
Women, n(%)	9640 (52.9%)	7020 (52.5%)	2620 (54.0%)	0.07	1886 (51.8%)	5871 (53.7%)	1883 (51.7%)	0.03
Education, n(%)				0.05				0.49
- Low	8060 (44.3%)	5983 (44.8%)	2077 (42.8%)		1600 (43.9%)	4830 (44.2%)	1630 (44.7%)	
- Intermediate	5409 (29.7%)	3948 (29.5%)	1461 (30.1%)		1121 (30.8%)	3234 (29.6%)	1054 (28.9%)	
- High	4743 (26.0%)	3433 (25.7%)	1310 (27.0%)		922 (25.3%)	2862 (26.2%)	959 (26.3%)	
APOE ε4 carrier status				< 0.001				0.06
- Non-carrier	13364 (73.4%)	13364 (100.0%)	0		2739 (75.2%)	7988 (73.1%)	2637 (72.4%)	
- ε4 heterozygote	4227 (23.2%)	0	4227 (87.2%)		784 (21.5%)	2572 (23.5%)	871 (23.9%)	
- ε4 homozygote	621 (3.4%)	0	621 (12.8%)		120 (3.3%)	366 (3.3%)	135 (3.7%)	
PRS	1.9 (0.2)	1.9 (0.2)	1.9 (0.2)	0.10	1.5 (0.1)	1.9 (0.1)	2.2 (0.1)	< 0.001
Incident AD, n(%)	374 (2.1%)	98 (0.7%)	276 (5.7%)	< 0.001	56 (1.5%)	215 (2.0%)	103 (2.8%)	< 0.001
Follow-up, years	12.8 (2.5)	12.9 (2.5)	12.7 (2.7)	< 0.001	12.8 (2.6)	12.9 (2.5)	12.8 (2.5)	0.40

**Table 2:** Plasma proteins associated with incident AD in the full sample (FDR < 0.05)

Name	Description	HR (95%CI) ^1^	FDR ^2^	LRT P ^2^^,^^3^	Reported times^4^
GFAP	Glial fibrillary acidic protein	2.35 (2.06, 2.67)	**1.32E-35**	**1.53E-02**	8
NEFL	Neurofilament light polypeptide	2.20 (1.81, 2.67)	**1.58E-12**	3.24E-01	8
SYT1	Synaptotagmin-1	1.62 (1.40, 1.89)	**1.50E-07**	3.32E-01	3
CEND1	Cell cycle exit and neuronal differentiation protein 1	1.35 (1.20, 1.51)	**2.08E-04**	9.76E-01	2
TGFBR1	TGF-beta receptor type-1	2.34 (1.67, 3.28)	**3.11E-04**	6.00E-01	4
MENT	Protein MENT	2.93 (1.86, 4.63)	**1.14E-03**	**6.02E-04**	2
APOE	Apolipoprotein E	0.57 (0.44, 0.73)	**1.57E-03**	5.53E-01	1
BCAN	Brevican core protein	0.57 (0.44, 0.74)	**7.66E-03**	9.37E-01	7

1. Models were adjusted for age, sex, education, and 20 PCs.

2. Bold values indicate FDR or likelihood ratio test (LRT) P value < 0.05.

3. APOE interaction was tested using LRTs comparing models with vs. without a protein × APOE interaction term; LRT P values are reported.

4. Reported by other studies. These studies were identified through a non-systematic review of the relevant literature.

## Data Availability

The UK Biobank data used in this study are accessible to verified researchers through an application to the UK Biobank (https://www.ukbiobank.ac.uk/) and were accessed under the corresponding approved application.
